# Psychosis in the Deaf Population: Prevalence, Phenomenology, and Diagnostic Challenges: A Narrative Review

**DOI:** 10.7759/cureus.96140

**Published:** 2025-11-05

**Authors:** Hooria Sarwar, Kareem Hamoudeh, Muzzamil Ahmadzada, Myra Dhingra, Elyse Batmaz, Manahil Adnan, Charissa Nichols, Jacqueline Seikunas

**Affiliations:** 1 Psychiatry, Rutgers University New Jersey Medical School, Newark, USA; 2 Psychiatry, Bergen New Bridge Medical Center, Paramus, USA; 3 Psychiatry, Stanford University, Palo Alto, USA; 4 Psychiatry, New York University, New York City, USA; 5 Psychiatry, Immaculate Heart Academy, Washington, USA; 6 Psychiatry, Scotch Plains High School, Scotch Plains, USA

**Keywords:** cultural competence, deaf, diagnostic challenges, hallucinations, language dysfluency, mental health disparities, psychosis, schizoaffective disorder, schizophrenia

## Abstract

Deaf and hard-of-hearing (DHH) individuals face unique challenges in psychiatric assessment, particularly in the diagnosis and treatment of psychotic disorders. Communication barriers, cultural misunderstandings, and clinician unfamiliarity with deaf language and norms may contribute to misdiagnosis, diagnostic uncertainty, and inadequate care. This narrative review synthesizes current literature on the prevalence, phenomenology, and diagnostic challenges of psychotic disorders in the deaf population, aiming to identify trends, gaps, and future research needs. A comprehensive search was conducted in three databases - PubMed, EBSCO Open Dissertations, and ScienceDirect - between April 1, 2025, and April 20, 2025. Keywords and MeSH terms related to psychosis, hallucinations, delusions, and deafness were used. Inclusion criteria encompassed peer-reviewed studies that investigated the prevalence, symptomatology, or diagnostic complexities of psychosis in DHH populations. Studies involving only hearing individuals, those without psychosis-related outcomes, or those lacking comparative or baseline data were excluded. Data were extracted independently by two reviewers and synthesized narratively. Nine studies were included, encompassing a total of 827 participants. The reported prevalence of psychotic disorders in deaf populations ranged from 28% to 58%. Schizoaffective disorder and schizophrenia were the most common diagnoses. Distinct phenomenological patterns emerged, with visual, tactile, and non-verbal auditory hallucinations frequently reported. High rates of diagnostic ambiguity, including overuse of psychotic disorder Not Otherwise Specified (NOS), were observed. Language dysfluency, cognitive impairment, and systemic barriers further complicated diagnostic accuracy. Psychotic disorders are prevalent in deaf psychiatric populations, yet cultural, linguistic, and systemic challenges often hinder assessment. Future research should focus on culturally adapted diagnostic tools, large-scale multisite prevalence studies, and qualitative investigations into deaf experiences of psychosis. Similar approaches should be extended to other sensory-impaired populations to uncover potential diagnostic disparities and inform equitable psychiatric care.

## Introduction and background

Approximately 15% of adults in the United States - around 37.5 million individuals aged 18 and over - report experiencing some degree of hearing difficulty [1]. The term “Deaf,” as defined by the National Deaf Center, is intentionally inclusive, encompassing individuals who identify as deaf, deafblind, deafdisabled, hard-of-hearing, late-deafened, or hearing-impaired [2]. While strides have been made in recognizing the deaf community as a distinct cultural and linguistic group, its mental health needs remain chronically underserved. Communication barriers, limited access to culturally competent care, and a legacy of marginalization contribute to significant disparities in psychiatric diagnosis and treatment. Among these, the experience of psychotic disorders in the deaf population is particularly underrepresented and misunderstood. Recent large-scale cohort data demonstrate that individuals with hearing loss have a substantially elevated risk of developing psychiatric disorders, approximately 2.5 times higher than those without hearing loss [3].

Psychotic disorders-such as schizophrenia, schizoaffective disorder, delusional disorder, and brief psychotic disorder-are defined by profound disturbances in perception, cognition, and behavior, often characterized by delusions, hallucinations, disorganized thought or speech, and negative symptoms such as blunted affect or diminished emotional expression [4]. Accurate diagnosis relies heavily on verbal communication, insight, and clinician interpretation, making the assessment of deaf individuals especially complex. Until the mid-1960s, deaf individuals with serious mental illness were institutionalized alongside hearing patients and treated by staff lacking proficiency in sign language [5]. Although the enactment of the Americans with Disabilities Act (ADA) markedly improved accessibility and awareness, significant barriers to mental health care for deaf individuals persist, often contributing to delayed diagnosis and suboptimal treatment outcomes. Cultural differences in communication, such as the use of American Sign Language (ASL), eye contact norms, and affective expression, are frequently misinterpreted as signs of disorganized thinking or psychosis. Access to mental health care for deaf individuals remains highly restricted, often leading to delayed diagnosis and treatment. Furthermore, the lack of trained clinicians proficient in sign language and deaf culture increases the risk of misdiagnosis and diagnostic ambiguity. These challenges highlight a critical gap in the literature and point to the need for multisite, population-based studies using native signers as diagnosticians to yield more reliable estimates and culturally valid clinical profiles.

This narrative review aims to synthesize current literature on psychosis in deaf individuals, with a focus on three central themes: (1) prevalence and patterns of psychiatric diagnoses among deaf patients; (2) the phenomenology of psychotic symptoms (i.e., the lived experience and presentation), including the types of hallucinations and delusions experienced; and (3) the diagnostic limitations and cultural misunderstandings that hinder accurate assessment and treatment. By integrating findings across institutional, clinical, and phenomenological studies, this review highlights the urgent need for culturally competent approaches and standardized diagnostic practices tailored to the deaf population.

## Review

Methods

A comprehensive literature search was conducted to identify studies examining the prevalence, phenomenology, and diagnostic challenges of psychosis in the deaf population. Three electronic databases -PubMed, EBSCO Open Dissertations, and ScienceDirect - were searched between April 1, 2025, and April 20, 2025. The search strategy utilized a combination of keywords and MeSH terms related to psychosis, deafness, prevalence, phenomenology, and diagnostic challenges. A detailed overview of the search terms and strategies is presented in Table 1.

**Table 1:** Search Strategy

Search Strategy	Databases/Registers	Number of Studies Before and After Filters	Filters Applied
(((("Schizophrenia Spectrum and Other Psychotic Disorders"[Mesh] OR "Psychotic Disorders"[Mesh]) OR ("schizophrenia"[Text Word] OR "schizoaffective"[Text Word] OR "schizophreniform"[Text Word] OR "schizotypal"[Text Word] OR "psychotic"[Text Word] OR "psychosis"[Text Word] OR "psychoses"[Text Word] OR "hallucination*"[Text Word] OR "delusion*"[Text Word])) OR (("Brief Psychiatric Rating Scale"[Mesh]) OR ("Clinician Rated Dimensions of Psychosis Symptom Severity"[Text Word] OR "Positive and Negative Syndrome Scale"[Text Word] OR "Brief Psychiatric Rating Scale"[Text Word] OR "Scale for the Assessment of Positive Symptoms"[Text Word] OR "Scale for the Assessment of Negative Symptoms"[Text Word]))) AND (("Deafness"[Mesh] OR "Deaf-Blind Disorders"[Mesh]) OR ("deaf*"[Text Word] OR "sensory depriv*"[Text Word]))) NOT ("deafferentation"[Text Word])	PubMed	624/7	Full-text, Clinical Study, Clinical Trial, Controlled Clinical Trial, Equivalence Trial, Observational Study, Randomized Controlled Trial, English, humans, exclude preprints
(schizophrenia OR psychosis OR hallucination OR delusion) AND (deaf OR "sensory deprivation") AND (scale OR rating) NOT deafferentation	ScienceDirect	4274/1616	Research article, English
(schizophrenia OR psychosis OR hallucination OR delusion) AND (deaf OR "sensory deprivation") AND (scale OR assessment OR rating) NOT deafferentation	EBSCO Open Dissertation	3/3	Dissertation

Eligibility Criteria

Eligibility criteria included peer-reviewed studies that examined psychosis in individuals who are DHH. Studies were included if they addressed prevalence, phenomenology, or diagnostic challenges related to psychotic disorders in this population. Both adult and pediatric populations were considered (Table 2).

**Table 2:** Inclusion and Exclusion Criteria RCT: randomized controlled trial; CCT: controlled clinical trial

Category	Inclusion Criteria	Exclusion Criteria
Population	Deaf or hard-of-hearing individuals, especially prelingually deaf adults and children (as defined by Clinical diagnosis or study description).	Studies involving only hearing individuals or those that do not differentiate between deaf and hearing participants.
Exposure/Prognostic Factor	Deafness (particularly prelingual), language deprivation, sensory deprivation, or use of culturally/linguistically appropriate psychiatric services.	Studies with no relevance to deafness or those that do not consider deaf-specific factors in psychiatric evaluation.
Outcomes	Outcomes related to prevalence, symptom presentation (e.g., hallucination type), diagnostic process, or treatment outcomes in deaf individuals with psychosis.	Studies that do not report outcomes relevant to psychosis, or focus on mental health outcomes unrelated to psychotic disorders.
Study Design	RCTs, CCTs, observational studies (cohort studies, case-control studies, cross-sectional studies), qualitative studies, and mixed-method studies.	Case reports, case series, reviews, editorials, commentaries, no comparison group/baseline data.
Publication Type	Peer-reviewed articles and trials (completed with results)	Non-peer-reviewed articles, conference abstracts/posters (without full data), trials (non-completed or completed without results).
Language	English	Other than English
Subjects	Humans	Animals

Studies were excluded if they did not involve DHH participants or failed to distinguish them from hearing individuals. Research focusing on mental health conditions unrelated to psychotic disorders, such as anxiety or depression alone, was omitted. Studies that lacked relevant outcomes related to prevalence, symptomatology, or diagnostic processes of psychosis were also excluded. Regarding study design, case reports, case series, commentaries, editorials, or studies without baseline or comparison data were not included. Non-peer-reviewed publications, including conference abstracts without full data or incomplete trials, were also excluded to maintain a focus on high-quality, relevant evidence.

Data Extraction and Management

A standardized template was used to extract key information from the included studies. This template recorded details such as author names, year of publication, study design, focus of the investigation, aims, and major findings. Two reviewers independently performed the data extraction. Disagreements were resolved through discussion, and when necessary, a third reviewer was consulted to ensure consensus.

Data Synthesis and Analysis

A narrative synthesis was used to analyze and summarize findings from the included studies, with a focus on the prevalence, symptomatology, and diagnostic challenges of psychosis in individuals who are DHH.

Results

**Figure 1:**
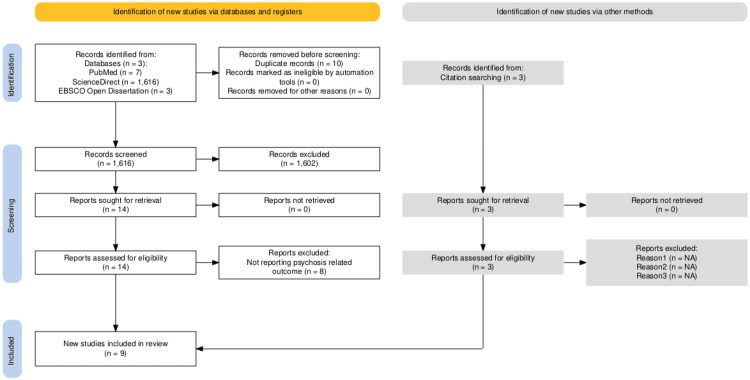
PRISMA flow diagram PRISMA: Preferred Reporting Items for Systematic Reviews and Meta-Analyses

To streamline the screening process, the Rayyan® application was utilized [6]. A comprehensive search across three electronic databases, PubMed, EBSCO Open Dissertations, and ScienceDirect, was performed using a combination of MeSH terms and text keywords related to psychosis, schizophrenia, hallucinations, delusions, and deafness, yielding a total of 4,901 records. After applying database-specific filters, this number was reduced to 1,626. Following the removal of 10 duplicate records, 1,616 unique articles were screened by title and abstract. Of these, 1,602 were excluded based on the predefined inclusion and exclusion criteria. Fourteen full-text articles were reviewed in detail, and eight were excluded for not reporting psychosis-related outcomes. An additional three studies were identified through citation searching. In total, nine studies were included in the final narrative review. This process is illustrated in Figure 1.

Summary of Included Studies

This review synthesizes findings from nine studies investigating psychosis in the deaf population, drawing on retrospective observational studies, chart reviews, qualitative analyses, and clinical case-control designs, with a total sample size of 827 participants, ranging from 10 to 544. The studies reveal wide variability in the prevalence of psychotic disorders, with schizophrenia and schizoaffective disorder frequently reported, and unique phenomenological features such as visual and tactile hallucinations are more common than in hearing populations. Several studies highlighted high rates of diagnostic uncertainty and misclassification, often due to language deprivation and limited clinician familiarity with deaf culture and communication. A summary of the included studies is detailed in Table 3.

**Table 3:** Summary of Included Studies NOS: Not otherwise specified: DHH: deaf or hard-of-hearing; SE: standard error

Author/Year	Country	Study Design	Population Characteristics	Aim of Study	Key Findings
Haskins 2004 [7]	USA	Retrospective Observational study	Inpatients at the specialized deaf psychiatric unit (n=43)	To assess diagnoses and cognitive/linguistic comorbidities in deaf psychiatric inpatients	62% had major mental illnesses, 58% had psychotic disorders, and 26% had a dual diagnosis with substance use. Diagnoses included schizophrenia (n=9), bipolar disorder (n=8), major depression (n=7), and schizoaffective disorder (n=3), totaling 27 patients (63%). Substance abuse was diagnosed in 22 patients (51%), with 11 (26%) having both mental illness and substance use. Additional diagnoses included intellectual disability (n=7, 16%) and borderline intellectual function (n=4, 9%). Other disorders were present in 17 patients (40%): anxiety disorders (n=8), pervasive developmental disorders NOS (n=4), depressive disorder NOS (n=3), and paraphilia (n=2).
Schonauer et al. 1998 [8]	Germany	Retrospective qualitative study	Prelingually deaf schizophrenic patients (n=67)	To examine the effect of deafness on hallucination	In the deaf sample, visual and auditory hallucinations were equally common in all but one case, where visual hallucinations were more frequent. Tactile hallucinations were reported by 15% of patients. A total of 23 patients reported hearing human voices, but only 11 could describe the content of what they heard. Additionally, 5 patients experienced non-verbal auditory hallucinations, such as noises or sounds. Hallucinatory visual communication, including seeing others signing or fingerspelling, was reported by 7 patients. Compared to hearing schizophrenic samples, deaf patients exhibited higher rates of visual and tactile hallucinations and fewer clearly defined auditory hallucinations.
Szymanek et al. 2007 [9]	Poland	Observational Study (Retrospective Chart Review)	10 patients with total (n=4) or partial (n=6) deafness hospitalized for psych issues	To explore the prevalence and clinical features of psychiatric disorders in deaf patients	Diagnoses included paranoid schizophrenia (n=2), depressive episode (n=2), organic catatonic disorder, organic personality disorder, delusional disorder, behavioral-emotional disorder, adaptation disorder, and mental retardation with alcohol abuse. Auditory hallucinations were reported in n=5 patients. Aggressive behavior was observed in 8 out of the 10 patients.
de Feu and McKenna 1999 [10]	UK	Cross-sectional Observation study	Prelingually deaf schizophrenic and schizoaffective patients (n=17)	To investigate the phenomenology of auditory hallucinations in profoundly deaf patients	Two patients described hearing voices through their hearing aids. Seven patients (41%) experienced non-verbal auditory hallucinations, five of whom also had verbal hallucinations. Visual hallucinations were reported by 9 patients (53%), somatic hallucinations by 8 (47%), and olfactory hallucinations by 3 (18%). Verbal auditory hallucinations were also reported by five patients who were profoundly deaf since birth. Across studies, the frequency of verbal auditory hallucinations in profoundly deaf schizophrenic patients has varied widely, from 12% to 83%, with this study showing a rate comparable to the 42% observed in hearing patients from the International Pilot Study of Schizophrenia.
Critchley et al. 1981 [11]	UK	Clinical study	Prelingually deaf schizophrenic patients (n=12)	To study hallucinations and delusions in deaf patients	Among 12 prelingually deaf schizophrenic patients, 10 (83%) reported visual hallucinations. Some were classical scenic hallucinations, while others were visual-verbal, resembling visual representations of verbal content.
Houston and Royse 1954 [12]	UK	Case-Control study	Deaf psychotic patients (n=40) vs hearing controls (n=40)	To compare the prevalence of paranoid symptoms in deaf vs hearing psychotic patients. Controlled for age and sex	In the experimental group of 40 deaf psychotic patients, 17 (42.5%) were classified as paranoid, compared to 8 (20%) in the matched control group of 40 hearing psychotic patients ( a significantly higher prevalence of paranoid symptoms among deaf patients (p = 0.026) was seen). A moderate but significant correlation between deafness and paranoid symptoms was found (tetrachoric correlation r ≈ 0.54, SE ≈ 0.14, Critical Ratio = 2.86, p < 0.01).
Black and Glickman 2006 [13]	USA	Retrospective Observational Study	Discharged adult patients at the Deaf Unit (n=64) vs control (n=180)	To compare deaf and hearing psychiatric inpatients on demographic, diagnostic, cognitive, language, and psychosocial functioning variables to better understand the clinical characteristics and needs of severely and chronically mentally ill deaf individuals.	In this study of deaf psychiatric inpatients (n = 64), psychotic disorders were diagnosed in only 28% (n = 18) of patients, compared to 88.9% (n = 160) of hearing inpatients (n = 180). Among deaf patients, specific psychotic diagnoses included schizoaffective disorder in 20.3% (n = 13) and schizophrenia in 6.3% (n = 4), as compared to non-hearing patients, among whom schizophrenia alone accounted for 47.7% (n = 86) and schizoaffective disorder for 37.7% (n = 68)
Landsberger and Diaz 2010 [14]	USA	Retrospective Observational Study	Adults deaf and severely hard-of-hearing (n=30); Hearing patients (comparison group) (n=60)	To compare the prevalence and diagnosis of psychotic disorders in deaf versus hearing psychiatric inpatients and explore diagnostic challenges related to language and cultural barriers.	Psychotic disorders were diagnosed in 43.3% of deaf patients (n = 13/30) and 61.7% of hearing patients (n = 37/60), but the difference was not statistically significant (p >.05). Among those with psychosis, 38% of deaf patients were diagnosed with psychotic disorder NOS, versus 3% of hearing patients. Schizoaffective disorder was diagnosed in 15% of psychotic deaf patients and 51% of psychotic hearing patients.
Pollard 1994 [15]	USA	Retrospective Cross-sectional observational study	A total of n = 84,437 public mental health case records were reviewed, of which n = 544 were identified as belonging to deaf DHH patients (adults and children)	To assess public mental health access, diagnostic patterns, and service disparities among deaf and hard-of-hearing individuals, with particular attention to how limitations in clinician expertise and system design may contribute to diagnostic challenges, including the misidentification of psychotic disorders.	Psychotic disorders were slightly higher in DHH patients (schizophrenia 8.2%, unclassified 3.5%) vs. the general sample (7.0%, 2.7%), but diagnostic uncertainty was greater with more deferred (4.4% vs. 1.5%), no diagnosis (4.1% vs. 1.4%), and missing data (17.5% vs. 12.4%) (p <.01/)

Discussion

The findings of this review reveal a complex and often fragmented understanding of psychosis in the deaf population. While existing literature has begun to shed light on the unique clinical and cultural considerations involved, significant variability in prevalence rates, symptom expression, and diagnostic practices highlights the challenges faced by both patients and clinicians. To meaningfully interpret these findings, the following sections discuss key patterns across studies, ranging from epidemiological trends and symptom phenomenology to diagnostic ambiguity, systemic barriers, and the need for culturally competent care. This discussion aims not only to synthesize what is currently known but also to critically examine the structural and conceptual gaps that continue to hinder accurate diagnosis and equitable treatment of deaf individuals experiencing psychosis.

Prevalence of Psychotic Disorders in Deaf Psychiatric Populations

Several studies have investigated the prevalence of psychotic disorders among the deaf psychiatric population, reporting a wide range of estimates. Haskins et al. [7] found that 58% of 43 deaf inpatients had psychotic disorders, including schizophrenia (n = 9) and schizoaffective disorder (n = 3). Black and Glickman [13] reported a lower prevalence of 28% among 64 deaf inpatients, with 20.3% diagnosed with schizoaffective disorder and 6.3% with schizophrenia, compared to 88.9% among hearing inpatients. In the study by Landsberger and Diaz [14], 43.3% of 30 deaf inpatients had psychotic disorders, compared to 61.7% of 60 hearing controls (p >.05); notably, 38% of psychotic deaf patients were diagnosed with psychotic disorder Not Otherwise Specified (NOS), compared to only 3% of hearing patients. Pollard found slightly higher rates of schizophrenia (8.2%) and unclassified psychosis (3.5%) in a large DHH sample (n = 544) compared to the general sample (7.0% and 2.7%, respectively), with significantly greater diagnostic uncertainty (deferred diagnosis: 4.4% vs. 1.5%; no diagnosis: 4.1% vs. 1.4%; missing data: 17.5% vs. 12.4%; p <.01) [15]. Szymanek et al. reported that among 10 deaf psychiatric inpatients, 2 were diagnosed with paranoid schizophrenia and 5 experienced auditory hallucinations [9].

Taken together, the prevalence of psychotic disorders in deaf individuals ranges from 28% to 58% across studies, with schizophrenia and schizoaffective disorder being the most commonly reported diagnoses. Some studies indicate higher prevalence among deaf populations compared to hearing controls, while others report similar or lower rates [7,13-15].

Overuse of Psychotic Disorder NOS and Diagnostic Uncertainty

This review highlights the diagnostic challenges in assessing psychosis among deaf individuals. Landsberger and Diaz [14] found that 38% of deaf inpatients with psychosis were diagnosed with the vague category of psychotic disorder NOS, compared to just 3% of hearing patients. Similarly, Pollard reported significantly higher diagnostic uncertainty in DHH patients, including more deferred diagnosis (4.4% vs. 1.5%), no diagnosis (4.1% vs. 1.4%), and missing data (17.5% vs. 12.4%) (p <0.01) [15]. Black and Glickman [13] observed reduced diagnostic clarity as well, with only 28% of deaf patients diagnosed with psychotic disorders compared to 88.9% of hearing inpatients, and a lower prevalence of schizophrenia among deaf individuals (6.3% vs. 47.7%). Together, these findings indicate a pattern of overreliance on nonspecific diagnoses and a greater degree of diagnostic ambiguity in the deaf psychiatric population. This pattern aligns with broader psychiatric debates on the use of NOS categories as diagnostic placeholders, which often emerge when cultural or linguistic barriers limit clinicians’ ability to apply standard diagnostic frameworks accurately.

Differences in Psychotic Subtype Diagnosis

Variations in the distribution of psychotic subtypes among deaf patients are evident across studies. Landsberger and Diaz [14] found that among deaf inpatients with psychosis, 15% were diagnosed with schizoaffective disorder, compared to 51% of hearing patients. Black and Glickman [13] reported that in their deaf sample, 20.3% were diagnosed with schizoaffective disorder and only 6.3% with schizophrenia, while in the hearing group, schizophrenia accounted for 47.7% and schizoaffective disorder for 37.7%. Similarly, Haskins et al. [7] noted that out of 25 deaf inpatients with psychotic disorders, 9 were diagnosed with schizophrenia and 3 with schizoaffective disorder. These findings suggest notable discrepancies in the pattern of psychotic subtype diagnoses between deaf and hearing individuals, with schizoaffective disorder often being more frequently assigned to deaf patients and schizophrenia more commonly diagnosed in hearing populations.

Phenomenology of Psychosis in Deaf Patients: Multimodal Hallucinations

Schonauer et al. [8] examined 67 prelingually deaf schizophrenic patients and found equal prevalence of visual and auditory hallucinations, with 15% experiencing tactile hallucinations and 7 reporting hallucinatory visual communication, such as seeing fingerspelling or signing. Although 23 patients reported hearing voices, only 11 could describe their content, and 5 experienced non-verbal auditory hallucinations like sounds or noises. In a separate study, Feu and McKenna [10] reported visual hallucinations in 53% of 17 profoundly deaf patients, somatic hallucinations in 47%, and olfactory hallucinations in 18%; 41% had non-verbal auditory hallucinations, and 5 reported verbal ones despite being deaf since birth. Critchley et al. [11] found that 83% of 12 prelingually deaf schizophrenic patients experienced visual hallucinations, including both scenic and visual-verbal types. Szymanek et al. [9] observed auditory hallucinations in 5 out of 10 deaf patients, most of which were verbal. These findings suggest that hallucinations in deaf patients often span multiple sensory modalities, with a strong presence of visual, tactile, and non-verbal auditory experiences.

Auditory Hallucinations in Prelingually Deaf Patients: Evidence and Interpretations

Feu and McKenna [10] reported that among 17 prelingually deaf schizophrenic and schizoaffective patients, 41% experienced non-verbal auditory hallucinations, such as drumming or indistinct speech sounds, and 5 patients reported verbal auditory hallucinations, despite being profoundly deaf since birth. Similarly, Schonauer et al. [8] found that of 67 prelingually deaf schizophrenic patients, 23 reported hearing human voices, although only 11 could articulate what was heard, and 5 experienced non-verbal auditory phenomena like noises or sounds. Szymanek et al. [9] observed that 5 of 10 deaf psychiatric inpatients also reported auditory hallucinations, most of which were verbal. These studies provide converging evidence that auditory hallucinations-both verbal and non-verbal-occur in prelingually deaf individuals, prompting important questions about their sensory nature and cognitive interpretation.

Visual and Tactile Hallucinations: Distinctive Features in Deaf Schizophrenia

Critchley et al. [11] reported that 10 out of 12 prelingually deaf schizophrenic patients experienced visual hallucinations, including both classical scenic hallucinations and visual-verbal imagery resembling signed language. Feu and McKenna [10] observed visual hallucinations in 53% and somatic (tactile) hallucinations in 47% of their 17 profoundly deaf participants, with additional reports of olfactory hallucinations in 18%. Similarly, Schonauer et al. [8] found visual and auditory hallucinations to be equally prevalent in their sample of 67 deaf schizophrenic patients, with 15% also experiencing tactile hallucinations. These findings suggest a distinctive pattern of multimodal hallucinatory experience in deaf individuals with schizophrenia, marked by a higher frequency of visual and tactile phenomena compared to hearing populations.

Delusional Patterns and Paranoid Presentations in Deaf Psychotic Patients

Delusional and paranoid presentations appear to be prominent features of psychosis in deaf individuals, raising important questions about how sensory experience and communication barriers may shape psychopathology. Houston [12] found a significantly higher prevalence of paranoid symptoms in deaf psychotic patients (42.5%) compared to matched hearing controls (20%) (p = 0.026), with a moderate but significant correlation between deafness and paranoia (r ≈ 0.54, SE ≈ 0.14, Critical Ratio = 2.86, p < 0.01). Haskins et al. [7] reported that among 43 deaf psychiatric inpatients, 58% were diagnosed with psychotic disorders, including nine cases of schizophrenia and three of schizoaffective disorder, suggesting a considerable burden of delusional and affective psychoses in this population.

Substance Use and Dual Diagnoses in Deaf Patients With Psychosis

Substance use and dual diagnoses are notably prevalent among deaf patients with psychosis, complicating their clinical profiles. Haskins et al. [7] reported that 26% of 43 deaf psychiatric inpatients had co-occurring substance use and mental illness, with 51% diagnosed with substance abuse overall. Szymanek et al. also noted the presence of comorbid mental retardation with alcohol abuse in a patient among their small cohort of 10 deaf individuals hospitalized for psychiatric issues [9]. These findings underscore the need to address substance use as a critical component in the assessment and treatment of psychosis within the deaf population.

Intellectual Disability and Cognitive Impairment as Diagnostic Confounders

Intellectual disability and cognitive impairment may act as diagnostic confounders in assessing psychosis among deaf individuals. Haskins et al. [7] reported that 16% of deaf inpatients were diagnosed with intellectual disability, with an additional 9% identified as having borderline intellectual function. Pollard similarly found higher rates of Axis II diagnoses, including mental retardation, among DHH patients compared to the general sample, alongside greater clinician difficulty in ruling out such conditions [15]. Szymanek et al. described at least one deaf patient with comorbid mental retardation and alcohol abuse in their study [9]. These findings suggest that cognitive limitations can obscure or complicate the recognition of psychotic symptoms, potentially leading to misdiagnosis or diagnostic uncertainty.

Language Dysfluency and Its Impact on Psychotic Symptom Interpretation

Language dysfluency has been shown to significantly impact the interpretation and diagnosis of psychotic symptoms in deaf individuals. Pollard highlighted that clinicians had more difficulty reaching definitive diagnoses for DHH patients, as reflected in higher rates of deferred (4.4% vs. 1.5%), missing (17.5% vs. 12.4%), and absent (4.1% vs. 1.4%) diagnoses compared to the general sample (p <0.01) [15]. Black and Glickman found that deaf patients were less likely to be diagnosed with psychotic disorders (28%) than hearing controls (88.9%), possibly due to challenges in distinguishing between language-based communication deficits and symptoms of psychosis [13]. Landsberger and Diaz further emphasized this diagnostic ambiguity, reporting that 38% of deaf patients with psychosis were labeled with psychotic disorder NOS, a vague classification used in just 3% of hearing patients [14]. These findings suggest that inadequate language proficiency, combined with limited clinician familiarity with deaf culture, may obscure the clinical picture and lead to misinterpretation or overgeneralization of symptoms.

Clinician Bias and the Misdiagnosis of Thought Disorder

Clinician bias may play a significant role in the misdiagnosis of thought disorders among deaf psychiatric patients. Landsberger and Diaz [14] reported that 38% of deaf inpatients with psychosis were diagnosed with psychotic disorder NOS, in stark contrast to just 3% among hearing patients. This disproportionate use of NOS suggests difficulty in accurately assessing symptoms, potentially stemming from clinicians’ limited understanding of deaf language and culture. Similarly, Black and Glickman [13] observed markedly lower rates of schizophrenia diagnoses among deaf inpatients (6.3%) compared to hearing inpatients (47.7%), indicating a possible under-recognition of formal thought disorder in deaf individuals. These findings imply that clinicians may mistake language dysfluency or atypical communication patterns for thought disorganization, leading to vague or incorrect diagnoses.

Systemic and Cultural Barriers to Accurate Diagnosis

Systemic and cultural barriers significantly hinder the accurate diagnosis of psychosis in deaf individuals. Pollard identified widespread diagnostic uncertainty among DHH patients, reflected in elevated rates of deferred (4.4% vs. 1.5%), missing (17.5% vs. 12.4%), and absent (4.1% vs. 1.4%) diagnoses compared to the general sample (p <.01), attributing these patterns to limitations in clinician expertise and service accessibility [15]. Landsberger and Diaz found that 38% of deaf patients with psychosis were labeled with psychotic disorder NOS, pointing to challenges in symptom interpretation arising from linguistic and cultural gaps [14]. Similarly, Black and Glickman reported dramatically lower psychotic diagnosis rates in deaf patients (28%) compared to hearing patients (88.9%), suggesting that institutional systems and a lack of deaf-aware training among professionals contribute to systemic underdiagnosis or misclassification [13].

Beyond diagnostic and systemic barriers, ethical considerations also intersect with these challenges. The use of interpreters in psychiatric settings raises issues of autonomy, privacy, and cultural representation, particularly when interpreters are not trained in mental health contexts. Furthermore, the systemic exclusion of deaf individuals from research design and clinical decision-making contributes to ongoing inequities in care. Incorporating deaf perspectives into study development and policy formation is, therefore, both an ethical and clinical imperative for achieving equitable psychiatric assessment and treatment.

The Need for Deaf-Centric Psychiatric Services and Competent Interpreters

The need for Deaf-centric psychiatric services and competent interpreters is emphasized across several studies. Black and Glickman highlighted that deaf psychiatric inpatients demonstrated markedly different diagnostic patterns compared to hearing peers, with significantly fewer diagnosed psychotic disorders, suggesting that standard clinical tools and communication methods may be insufficient [13]. Pollard attributed higher rates of diagnostic uncertainty in DHH patients to a lack of specialized services and clinician training, leading to frequent misinterpretations and gaps in care [15]. Similarly, Landsberger and Diaz reported that 38% of deaf patients with psychosis were diagnosed with psychotic disorder NOS, a vague classification often used when effective communication and culturally informed assessments are lacking [14]. These findings collectively underscore the urgent need for psychiatric settings equipped with deaf-aware clinicians and interpreters trained in mental health to improve diagnostic precision and patient outcomes.

Strengths and Limitations of the Review and Included Studies

This review provides a valuable synthesis of a niche yet critically important topic by systematically analyzing psychosis in deaf populations through the lens of prevalence, phenomenology, and diagnostic challenges. A notable strength lies in its structured search strategy and focus on culturally specific diagnostic complexities often overlooked in mainstream psychiatric research. The inclusion of studies from multiple countries adds cross-cultural depth, and the discussion highlights systemic and clinical barriers with practical implications for psychiatric care. A narrative review design was selected because the existing literature on psychosis in deaf populations is limited, methodologically heterogeneous, and predominantly qualitative, precluding the use of systematic review or meta-analytic methods.

However, the review and included studies are not without limitations. The studies examined vary widely in sample size, methodology, and geographic scope, resulting in significant heterogeneity that limits the generalizability of findings across the broader deaf and DHH population. Although pediatric populations were eligible for inclusion, the majority of studies focused on adults, limiting the ability to generalize findings to deaf children and adolescents. Additionally, ethical considerations-such as patient autonomy in the use of interpreters, cultural misrepresentation, and systemic exclusion-were underexplored. Finally, the lack of participatory research involving deaf individuals limits the ability of current studies to authentically represent the lived experiences of this underserved population.

Future research direction

Based on the findings and gaps identified in this review, several future research directions emerge. First, large-scale, multicenter studies are needed to better estimate the true prevalence of psychotic disorders in deaf populations, using standardized diagnostic criteria and culturally adapted assessment tools. These studies should aim to include more diverse samples in terms of age, geographic location, and cultural context to enhance generalizability. Second, research should focus on the development and validation of diagnostic instruments specifically tailored for DHH individuals, particularly those who are prelingually deaf or language dysfluent. Third, qualitative studies exploring the subjective experience and interpretation of hallucinations and delusions in deaf individuals can provide deeper insights into their unique phenomenology and lived experience. Fourth, further investigation into clinician bias and diagnostic accuracy, especially regarding the overuse of psychotic disorder NOS, is needed to assess how communication and cultural barriers influence clinical judgment. Fifth, intervention-based studies evaluating the impact of Deaf-centric services, including access to trained mental health interpreters and deaf-aware clinicians, are essential to determine how structural changes improve diagnostic precision and outcomes. Additionally, there is a critical need for studies specifically focused on pediatric deaf populations, as children and adolescents remain significantly underrepresented in current research. Ethical dimensions, such as the role of informed consent, interpreter use, and autonomy, should also be more systematically addressed in future work. Finally, similar research should be extended to other populations with sensory or communication disabilities, such as the visually impaired, to explore whether comparable diagnostic disparities and challenges exist across different disability groups.

## Conclusions

This review highlights the prevalence, symptom patterns, and diagnostic challenges of psychosis in the deaf population, synthesizing findings from diverse study designs. Schizophrenia and schizoaffective disorder appear common among deaf psychiatric inpatients, though reported rates vary widely. Multimodal hallucinations, especially visual and tactile, are frequently observed, and the occurrence of auditory hallucinations in prelingually deaf individuals challenges conventional assumptions about sensory processing in psychosis. Across studies, diagnostic ambiguity was common, with a disproportionate use of psychotic disorder NOS and higher rates of deferred or absent diagnoses, underscoring systemic and cultural barriers such as clinician unfamiliarity with deaf culture, language dysfluency, and misinterpretation of culturally normative behaviors. These findings underscore the urgent need for culturally and linguistically adapted diagnostic frameworks, validated assessment tools for deaf patients, and improved access to Deaf-centric psychiatric care. Future research should prioritize large-scale, multisite studies using standardized instruments, as well as qualitative approaches, to better understand the lived experiences of deaf individuals with psychosis.
